# The connection of dysfunctional breathing with Self-Government styles in the Russian population during the COVID-19 pandemic

**DOI:** 10.1192/j.eurpsy.2022.962

**Published:** 2022-09-01

**Authors:** J. Koniukhovskaia, E. Pervichko, O. Mitina, O. Stepanova, V. Petrenko, I. Shishkova, E. Dorokhov

**Affiliations:** 1 Pirogov Russian National Research Medical University, Clinical Psychology Department, Moscow, Russian Federation; 2 Lomonosov Moscow State University, Psychology, Moscow, Russian Federation; 3 Ryazan State Medical University named after I.P. Pavlov, Faculty Of Clinical Psychology, Ryazan, Russian Federation

**Keywords:** Covid-19 pandemic, dysfunctional breathing, Self-Government styles

## Abstract

**Introduction:**

Dysfunctional breathing is experienced as “difficulty in inhaling” and is similar to the symptoms of COVID-19 (Gavriatopoulou et al., 2020), which justifies the relevance of studying this phenomenon in the conditions of the COVID-19 pandemic.

**Objectives:**

To identifiy a relationship between self-management styles and the severity of dysfunctional breathing in the uninfected COVID-19 population of Russia.

**Methods:**

The author used the socio-demographic questionnaire, the Naimigen Questionnaire (Van Dixhoorn, Duivenvoordent, 1985) and J. Kuhl’s and A. Fuhrman’s Self-Government Test (Kool, Furman, 1998; Kul, Kvirin, Kool, 2020). The study was conducted online from April 27 to December 28, 2020. It was attended by 1,362 people from all regions of Russia (38.3 ±11.4y.o.).

**Results:**

The components are Self-regulation (r = -0.454, p = 0.000) and Self-Control (r =-0.197, p=0.000). There is also a component of Will Development (r=-0,297, p = 0,000) and Sensitivity to oneself (r=-0,480, p=0,000). It is important to note that dysfunctional breathing has a strong positive correlation with the component of life stress experiencing (=0.335, p=0.000). At the same time, the components of Self-regulation and Self-sensitivity have large correlation coefficients, which indicates their greater role.

**Conclusions:**

People with low self-regulation and self-control, as well as with less expressed will and sensitivity to themselves, are more likely to have dysfunctional breathing and a more pronounced experience of life stress in a pandemic. The described components can be used as “targets” for individualized psychotherapy of dysfunctional breathing in the conditions of the COVID-19 pandemic. The study was supported of the Russian Science Foundation, project No. 21-18-00624.

**Disclosure:**

The study was carried out with the support of the Russian Science Foundation, project No. 21-18-00624.

